# Silenced LINC01134 Enhances Oxaliplatin Sensitivity by Facilitating Ferroptosis Through GPX4 in Hepatocarcinoma

**DOI:** 10.3389/fonc.2022.939605

**Published:** 2022-07-08

**Authors:** Xiaofeng Kang, Yan Huo, Songhao Jia, Fuliang He, Huizi Li, Qing Zhou, Nijia Chang, Donghui Liu, Rongkuan Li, Yi Hu, Ping Zhang, An Xu

**Affiliations:** ^1^Department of Oncology, The Second Medical Center & National Clinical Research Center for Geriatric Diseases, Chinese PLA General Hospital, Beijing, China; ^2^International School of Public Health and One Health, Hainan Medical University, Hainan, China; ^3^Department of Ophthalmology, PLA Rocket Force Characteristic Medical Center, Beijing, China; ^4^Department of Infectious Disease, the Second Affiliated Hospital of Dalian Medical University, Dalian, China; ^5^Department of Liver Research Center, Beijing Friendship Hospital of Capital Medical University, Beijing, China; ^6^Department of gastroenterology, Second Medical Center of Chinese PLA General Hospital, Beijing, China; ^7^School of Traditional Chinese Medicine, Beijing University of Chinese Medicine, Beijing, China; ^8^State Key Laboratory of Pathogen and Biosecurity, Beijing Institute of Microbiology and Epidemiology, Beijing, China

**Keywords:** linc01134, Nrf2, GPx4, OXA, ferroptosis, HCC cancer

## Abstract

**Purpose:**

Recently, long noncoding RNA LINC01134 has been shown to reduce cell viability and apoptosis *via* the antioxidant stress pathway, thereby enhancing OXA resistance in hepatocellular carcinoma. However, the association of LINC01134 with ferroptosis and the underlying molecular mechanisms remain to be elucidated.

**Methods:**

Bioinformatics analysis was employed to screen lncRNAs positively correlated with GPX4 and poor clinical prognosis. And Western blot and RT-PCR analysis in HCC cells confirmed the effect of LINC01134 on GPX4 expression. In addition, LINC01134 siRNA was transfected in HCC cells to detect the changes in cell viability, ROS, lipid peroxidation, MDA levels and GSH/GSSG levels. CCK-8, colony formation and apoptosis assays were performed to determine the effect of LINC01134 on cell death. The effect of LINC01134 and OXA on Nrf2 transcriptional binding to GPX4 was analyzed using dual luciferase reporter assay and CHIP. The expression of GPX4 and Nrf2 in HCC tissues was detected by FISH and IHC.

**Results:**

LINC01134 is a novel lncRNA positively correlated with GPx4 and associated with poor clinical prognosis. Silenced LINC01134 conferred OXA sensitivity by enhancing total ROS, lipid ROS, MDA levels and decreasing GSH/GSSG ratio. Mechanistically, LINC01134 and OXA could promote Nrf2 recruitment to the GPX4 promoter region to exert transcriptional regulation of GPX4. Clinically, LINC01134 was positively correlated with GPX4 or Nrf2, demonstrating the clinical significance of LINC01134, Nrf2 and GPX4 in OXA resistance of HCC.

**Conclusions:**

We identified LINC01134/Nrf2/GPX4 as a novel and critical axis to regulate HCC growth and progression. Targeting GPX4, knocking down LINC01134 or Nrf2 could be a potential therapeutic strategy for HCC.

## Introduction

Hepatocellular carcinoma is the second major cause of cancer-related mortality as the most frequent liver cancer (1). Oxaliplatin is the first chemotherapy drug approved for advanced HCC worldwide, which is one of the commonly used chemotherapeutics in HCC (2). Oxaliplatin causes DNA damage and tumor cell apoptosis through the connection of platinum atoms and base G on the DNA chain (3). OXA is now one of the most effective systemic chemotherapeutic agents for HCC treatment, but the emergence of OXA resistance is also one of the key factors responsible for the failure of cancer treatment (4). In addition, multiple articles reported that ferroptosis was closely associated with OXA sensitivity in tumors (5, 6). Therefore, investigating the potential mechanisms and developing effective anti-OXA resistance strategies have essential significance in clinical priorities.

LncRNAs are defined as transcripts of more than 200 nucleotides, which played widespread roles in gene regulation and other cellular processes (7). LncRNAs have been reported to perform various functions including organization of nuclear domains, regulation of RNA or proteins molecules and cis or trans transcriptional regulation (7). In addition, analysis of transcriptome profiles over the past few years has shown that plenty of lncRNAs are related to diverse cancers (8). Research have reported that LncRNA LUCAT1 is highly expressed in liver cancer and other malignant tumors, which regulates tumor proliferation, invasion, and migration *via* various mechanisms (9). LncRNA HOTAIR is remarkably up-regulated in breast cancer patients and its expression strongly predicts cancer metastasis and lethal (10). Moreover, excessive LINC00336 facilitates cell growth, tumorigenesis, and suppresses ferroptosis through serving as a competing endogenous RNA (11). Homo sapiens long intergenic non-protein-coding RNA 1134 (LINC01134), acting as a long non-coding RNA producing a 1,960 bp transcript, was recently shown to boost the occurrence and development of HCC and was reported to be a poor sign of survival in HCC patients (12, 13). In our previous study (14), we found that LINC01134 confers oxaliplatin resistance by facilitating p62 Transcription in HCC, however, the effect of LINC01134 on oxaliplatin resistance failed to be completely abolished when p62 was knocked out, suggesting that there must be some other mechanisms mediating the effect of LINC01134 on oxaliplatin resistance, which needs further investigation.

Ferroptosis is a lipid peroxidation and iron dependent new mode for regulatory cell death, which is different from cell necrosis, autophagy and apoptosis (15). Ferroptosis plays a vital regulatory role in chemotherapy tolerance. Yu et al. reported the ferroptosis-specific inducer erastin significantly enhances the anti-cancer activity of the first-line chemotherapeutic drugs cytarabine and doxorubicin in HL60 cells (16). Glutathione peroxidase 4 (GPX4) acting as the major protective mechanism of membrane peroxidation which derived from cystine/glutamate-cysteine ligase (GCL)/glutathione (GSH), is a critical suppressor of ferroptotic cell death (17). Our results established that LINC01134 positively regulates GPX4 through transcription factor Nrf2. Silenced LINC01134 induces ferroptosis of liver cancer cells and reduces the resistance to oxaliplatin, which provides a new basis for targeted therapy of liver cancer.

## Results

### LINC01134 Is a Novel GPX4 Positively Correlated lncRNA and Associated With Poor Clinical Prognosis

**Figure 1:**
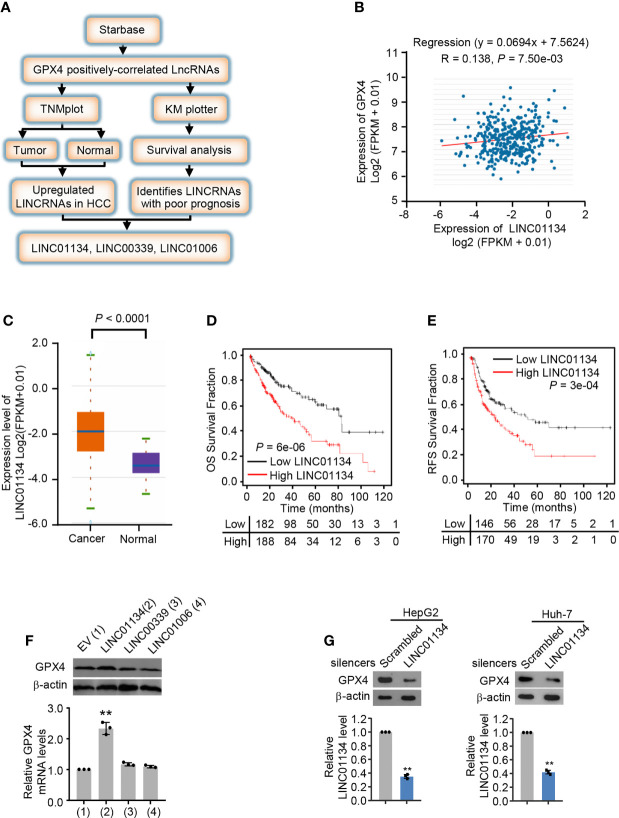
LINC01134 is a novel GPX4 positively correlated LINCRNA and associated with poor clinical prognosis. **(A)** Schematic diagram of the screening process for identifying up-regulated LINCRNAs in HCC and positively related to GPX4. **(B)** Pearson’s correlation analysis of LINC01134 and GPX4 expression in liver cancer tissues (http://starbase.sysu.edu.cn/). **(C)** The LINC01134 expression levels between cancer tissues and normal tissues were compared by TNM plot (https://www.tnmplot.com/). **(D, E)** Kaplan-Meier analysis of the recurrence-free survival and overall survival rate of HCC patients with high or low LINC01134 expression (http://kmplot.com/analysis/). **(F)** The mRNA and protein levels of GPX4 in HepG2 cells transfected with empty vector, LINC01134, LINC00339 and LINC01006 expression vectors. **(G)** The expression levels of GPX4 and LINC01134 in HepG2 (left panel) and Huh-7 (right panel) cells transfected with scrambled and LINC01134 silencers. The data are expressed as mean ± standard error. Data are shown as the mean ± SEM; n ≥ 3 independent experiments, two-tailed Student’s t-test: **P < 0.01.

To investigate the new lncRNAs positively regulating GPX4, we analyzed three HCC databases by the screening strategy revealed in **Figure 1A**. Briefly speaking, we first analyzed GPX4 positively-correlated lncRNAs from Starbase database (http://starbase.sysu.edu.cn/), and then we screened lncRNAs highly expressed in cancer tissues compared with normal tissues in TNMplot (https://tnmplot.com/analysis/), and investigated the survival of lncRNAs in Kaplan Meier plotter (http://kmplot.com/analysis/). Finally, we identified three lncRNAs, LINC01134, LINC00339 and LINC01006 positively correlated with GPX4 and demonstrate poor clinical prognosis (**Figures 1B-E** and **Figures S1A-H**). In order to verify whether the screened lncRNAs could up-regulate the expression of GPX4 in fact, we transfected the three lncRNAs into HepG2 and Huh-7 cells, respectively. The results demonstrated only LINC01134 caused a significant increase in GPX4 mRNA and protein levels in both HCC cells (**Figure 1F** and **Figure S1I**). In order to further verify the role of LINC01134 in HCC cells, we knocked down LINC01134 with the specific smart pool of silencers and discovered the expression level of GPX4 was obviously decreased in the two HCC cells lines (**Figure 1G**). These data demonstrate that LINC01134 positively correlated the GPX4 expression in HCC cells.

### Silenced LINC01134 Enhances the Sensitivity of Ferroptosis Inducers

**Figure 2:**
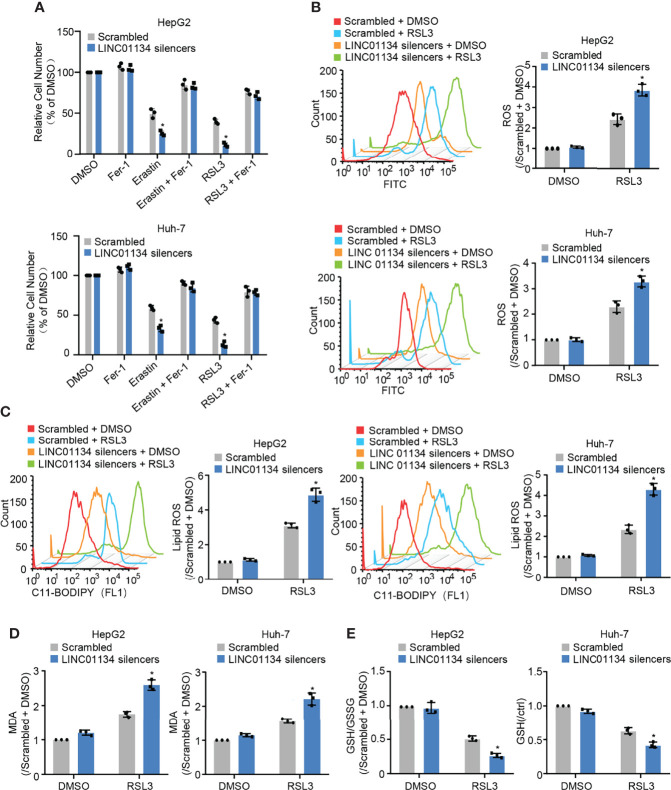
Silenced LINC01134 enhances sensitivity of HCC cells to OXA and promotes RSL3-induced ferroptosis. **(A)** Huh-7 and HepG2 cells were transfected with scrambled or LINC01134 silencers with or without treatment of RSL3 (2 μM), erastin (10 μM), and ferrostatin (2 μM). The histogram shows relative cell number measured by CCK-8 assays. **(B-E)** HepG2 and Huh-7 cells were transfected with scrambled or LINC01134 silencers with or without RSL3 (2 μM). Levels of total ROS **(B)**, lipid ROS **(C)**, Lipid Oxidation (MDA) **(D)** and GSH/GSSG **(E)** were analyzed. Data are shown as the mean ± SEM; n ≥ 3 independent experiments, two-tailed Student’s t-test: *P < 0.05.

GPX4 can directly restrict lipid peroxides to prevent ferroptosis, serving as a typical intracellular antioxidant enzyme (18). GPX4 has been reported as a suppressor of RSL3- and erastin-induced ferroptosis (19). Results show that silenced LINC01134 increased RSL3- and erastin-induced cell death, and compared with erastin, RSL3 has the most obvious inhibitory effect (**Figure 2A** and **Figures S2B, C**). Also, silenced LINC01134 increased the levels of total ROS, lipid ROS and one of the detection indicators of ferroptosis, PTGS2 (**Figures 2B, C** and **Figure S2A**). Knockdown of LINC01134 also raised the MDA concentration and decreased the ratio of GSH/GSSG induced by RSL3 (**Figures 2D, E** and **Figures S2D, E**). Together, our results demonstrate that silenced LINC01134 obviously enhances the sensitivity of RSL3.?>

### Silenced LINC01134 Downregulates OXA Resistance Through GPX4 Pathway

**Figure 3:**
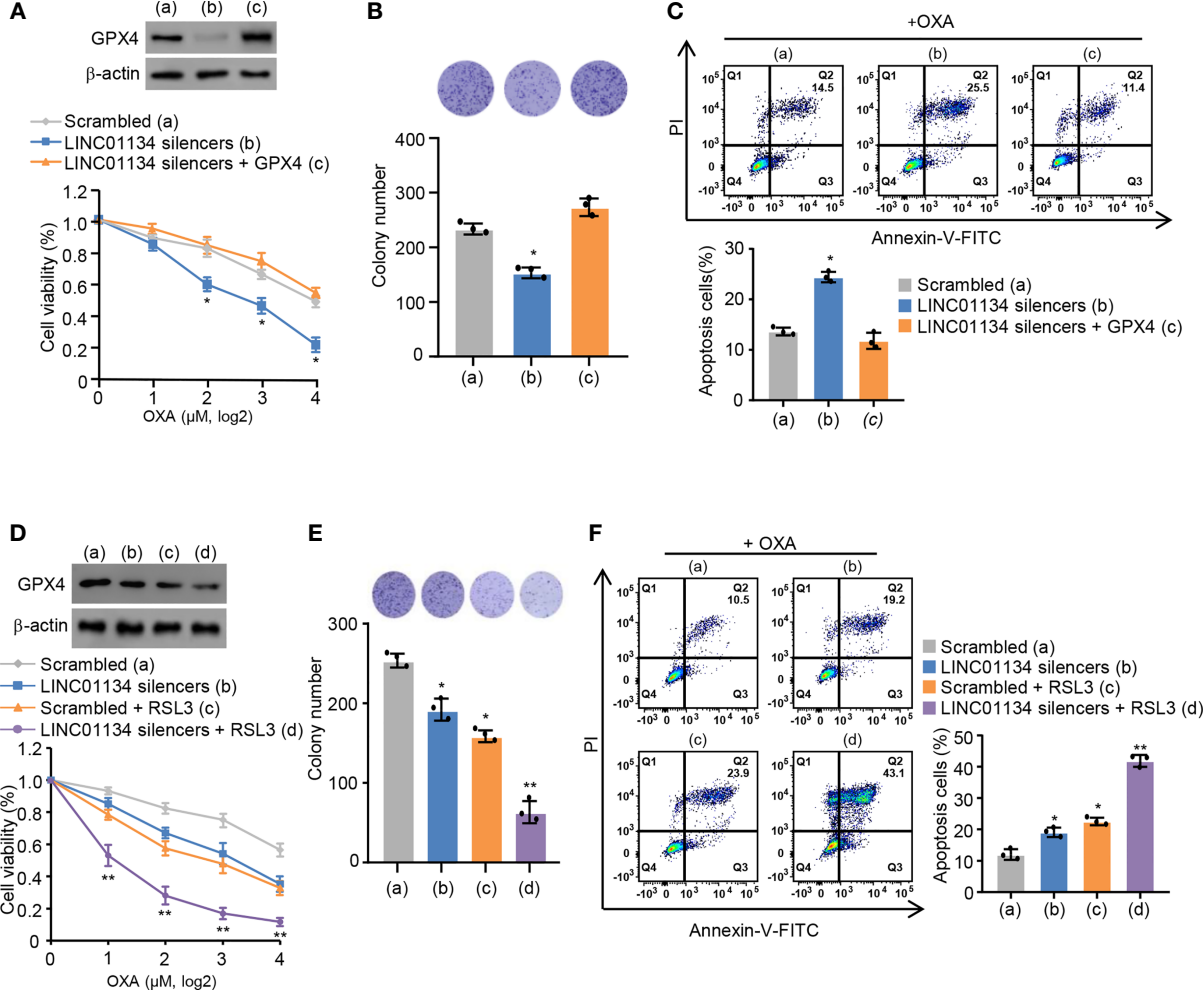
Silenced LINC01134 enhances sensitivity of HCC OXA resistance through GPX4 pathway. **(A)** HepG2 cells were transfected with scrambled, LINC01134 silencers, or LINC01134 silencers plus pcDNA3.0-GPX4. Relative cell number was tested *via* CCK-8 assays. Representative immunoblot indicates GPX4 expression. **(B)** HepG2 cells were transfected with scrambled, LINC01134 silencers, or LINC01134 silencers plus pcDNA3.0-GPX4. Representative image of colony formation assay shows the colonies in dishes. The histogram indicates colony number. **(C)** Representative flow cytometry analysis of FITC/PI staining of **(A)** with OXA (8 μM, 3 days) reflects the cell death rate. **(D)** HepG2 cells were transfected with scrambled, LINC01134 silencers, with or without RSL3 treatment. Relative cell number was determined tested *via* CCK-8 assays. Representative immunoblot indicates GPX4 expression. **(E)** HepG2 cells were transfected with scrambled, LINC01134 silencers, with or without RSL3 treatment. Representative image of colony formation assay indicates the colonies in dishes. The histogram shows colony number. **(F)** Representative flow cytometry analysis of FITC/PI staining of **(C)** with OXA (8 mM, 3 days) indicates the cell death rate. Data are shown as the mean ± SEM; n ≥ 3 independent experiments, two-tailed Student’s t-test: *P < 0.05, **P < 0.01.

Ma et al. previously reported LINC01134 augments OXA resistance *via* decreasing cell viability and apoptosis by the anti-oxidative stress pathway in HCC (14). It is reported that ferroptosis is a regulated form of cell death relying on oxidative stress (20). Therefore, we speculate whether LINC01134 may regulate OXA resistance through ferroptosis. Viability assays, colony formation assays, and apoptosis assays demonstrated the sensitization caused by LINC01134 knockdown could be reversed *via* GPX4 re-expression (**Figures 3A-C** and **Figure S3A**) and increased by RSL3 (**Figures 3D-F** and **Figure S3B**). In a word, our data demonstrates silenced LINC01134 decreases OXA resistance by reducing the cell viability, colony formation number and increasing cell death *via* the GPX4 pathway in HCC cells. To test whether LINC01134 is specific for chemotherapy in HCC, we did assays for sensitivity to tyrosine kinase inhibitors (TKIs) and found that LINC01134 could also mediate sensitivity to sorafenib (**Figures S3C, D**).

### LINC01134 Up-Regulates the Expression of GPX4 *Via* Augmenting the Transcription Factor Nrf2 Binding Onto GPX4 Promoter

**Figure 4:**
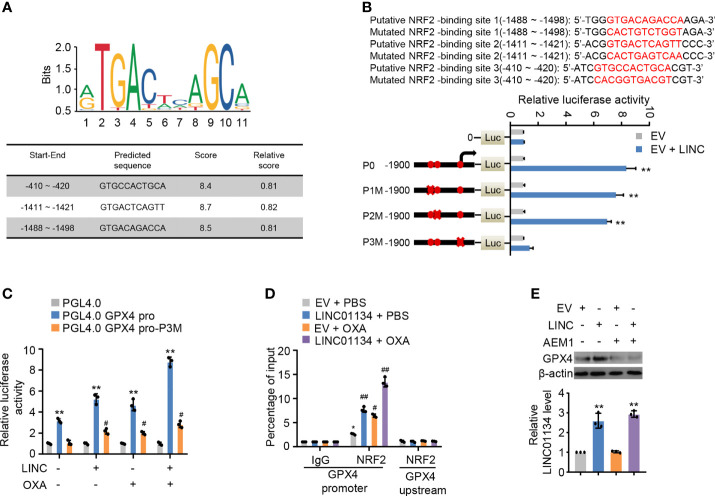
LINC01134 promotes GPX4 expression through Nrf2 transcription. **(A)** Motif analysis of Nrf2 binding peaks to GPX4 promoter sequence according to JASPER website (http://jaspar.genereg.net/). **(B)** Luciferase activity of different segmented GPX4 promoters of HepG2 cells transfected with indicated plasmids. Solid circles indicate the putative location of the Nrf2 binding sites, and the “X” indicates the mutated Nrf2 binding sites. The red letters in each bound area show the putative or mutated Nrf2 binding sequences. **(C)** Luciferase assays for the selected GPX4 promoter reporters from **(A)** transfected with LINC01134 and treated or not with OXA. **(D)** ChIP analysis for Nrf2 occupancy on the GPX4 the promoter upstream or promoter in HepG2 cells transfected with LINC01134 and treated or not with OXA. **(E)** Representative immunoblots indicate the GPX4 expression in HepG2 cells transfected with EV or LINC01134 and treated with AEM1 (5 μM, 1 day). The histogram indicates the LINC01134 expression in HepG2 cells. All data are presented as Mean ± SD.; two-tailed unpaired Student’s t-test: *P < 0.05, **P < 0.01, ^#^P < 0.05, ^##^P < 0.01.

Study has shown LINC01134 was mainly localized to the nucleus, with some localization in the cytoplasm (14). Consequently, we inferred LINC01134 increased GPX4 transcription levels mediated *via* transcriptional factors. GPX4 has been reported to be an established transcriptional target of transcriptional factor Nrf2 (21), which transcriptionally regulated a large fraction of genes thus far related to ferroptosis in the context of ferroptosis. To further determine if LINC01134 increases GPX4 expression by Nrf2 in HCC cells, three putative sequences of Nrf2 binding to the GPX4 promoter were provided by the JASPAR website (http://jaspar.genereg.net/). Analysis of several GPX4 promoter mutation reporter gene constructs illustrated a LINC01134 over-expression inhibitory element was located in the promoter region of -410 to -420 bp (**Figures 4A, B**). Over-expression of LINC01134 activated the GPX4 promoter activity containing the third putative Nrf2-binding site, but not the third mutated Nrf2-binding site (**Figure 4C**). Moreover, abnormally expressed LINC01134 enhanced the recruitment of Nrf2 to GPX4 promoter, and OXA treatment further increased the effect of recruitment (**Figure 4D** and **Figure S5A**). The specific Nrf2 inhibitor AEM1 inhibited the binding of Nrf2 to LINC01134 promoter region (**Figure S5B**). Inhibition of Nrf2 with AEM1 reduced the expression of GPX4. Importantly, the suppression of Nrf2 almost abrogated the ability of LINC01134 to active the expression of GPX4 (**Figure 4E**). In addition, knockdown of LINC01134 or treatment of AEM1 resensitized OXA-resistant cell lines to OXA (**Figure S5C**). In short, the above data collectively shows LINC01134 up-regulates the expression of GPX4 *via* promoting transcriptional factor Nrf2 binding onto GPX4 promoter by interaction of specific RNA-DNA sequence.

### Clinical Significance of LINC01134/Nrf2/GPX4 Axis in Hepatocellular Carcinoma

**Figure 5:**
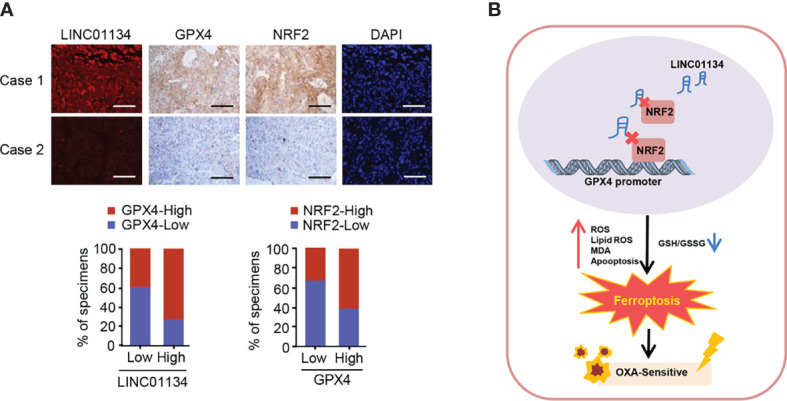
The relevance between the LINC01134 and GPX4 expression and the relationship between Nrf2 and GPX4 in liver cancer patients. **(A)** FISH staining for LINC01134 and representative IHC staining for GPX4 and Nrf2 in HCC patients with high or low LINC01134 expression. Scale bar, 50 μm. **(B)** Suggested pattern diagram for the function of the LINC01134 in oxaliplatin resistance by regulating ferroptosis of HCC cells.

TCGA database analysis demonstrates that GPX4 predicts poor prognosis in HCC (**Figure S5D**). In order to determine whether the LINC01134/Nrf2/GPX4 axis has clinical correlation and pathological relationship with the occurrence of HCC, we examined the expression of LINC01134 by FISH, and the expression of GPX4 and Nrf2 *via* IHC in a group of hepatocellular carcinoma tissues (n = 58). The GPX4 and Nrf2 antibodies specificity were certified (**Supporting Figure S4**). The Nrf2-low and LINC01134-low group displayed the lower expression of GPX4, but the Nrf2-high and LINC01134-high group displayed the higher expression of GPX4 (**Figure 5A**). In a word, the above data summarize the clinical relevance of LINC01134/Nrf2/GPX4 axis in oxaliplatin resistance in HCC.

## Discussion

Liver cancer, as the most frequent cause of cancer deaths worldwide, is the only one of the top five deadliest cancers to have an annual percentage growth in occurrence (22). For patients with advanced HCC, until recently, tyrosine kinase inhibitors (such as sorafenib and lenvatinib) were the only licensed systemic therapy (23–25), and immune checkpoint inhibitors (such as nivolumab and multuzumab) has been approved by several regulatory agencies as a second-line treatment after sorafenib (26–28), but oxaliplatin (OXA) remains one of the major systemic chemotherapy drugs and can be used as follow-up and maintenance treatment for patients with advanced hepatocellular carcinoma (29). A previous prospective, randomized, controlled, international multicenter clinical phase III study in China confirmed the efficacy and safety of OXA based FOLOFX4 (infusional fluorouracil, leucovorin, and oxaliplatin) in the treatment of HCC patients in Asia Pacific region, especially in China (30). Regimens including FOLFOX and gemcitabine plus cisplatin can be used in sorafenib refractory patients with good performance status if no clinical trials are available (31). In 2021, Li et al. also found that FOLFOX-HAIC (hepatic arterial infusion chemotherapy with infusional fluorouracil, leucovorin, and oxaliplatin) significantly improved the overall survival of patients with unresectable large HCC compared with TACE (transarterial chemoembolization) (32). However, OXA resistance in HCC is a main problem, and there is an urgent need to improve the response to this chemotherapy drug (33). Our work identified LINC01134/Nrf2/GPX4 as a key axis regulating OXA resistance in HCC (**Figure 5B**).

Zhang et al. has reported that LINC01134 can activate the transcription of AKT1S1 by binding to the promoter of AKT1S1, further activating NF- κB signaling pathway, thereby playing a role in promoting the migration and invasion of HCC cells (10). Interestingly, we found that LINC01134 is a novel lncRNA positively related to GPX4 and associated with poor clinical prognosis in HCC, which is consistent with previous findings. Besides that, we reported for the first time that silenced LINC01134 contributes to OXA sensitivity by inducing ferroptosis in HCC, characterized by enhancement of the total ROS, lipid ROS, MDA, and reduction of GSH/GSSG ratio. Mechanistically, LINC01134 upregulates the expression of GPX4 *via* strengthening the binding of transcription factor Nrf2 onto the GPX4 promoter. Clinically, LINC01134 is positively associated with GPX4 or Nrf2, demonstrating the clinical significance of LINC01134, Nrf2 and GPX4 in OXA resistance of HCC. Therefore, we may improve the sensitivity to OXA by inhibition of the expression of LINC01134 or GPX4 as we presented in this study. Detection of LINC01134 expression may be a promising strategy to evaluation the efficacy of OXA in HCC patients.

Current treatment regimens commonly used in the clinic of liver cancer chemotherapeutic drugs or molecularly targeted agents are prone to the phenomenon of drug resistance, and it is crucial to study their resistance mechanisms (34, 35). Previous studies showed that overexpression of miR-4277 in HCC cells reduced the elimination of sorafenib in HCC cells and enhanced the sensitivity of HCC cells to sorafenib by inhibiting the expression of CYP3A4 (36). Li et al. found that Rhamnetin treatment decelerated the metabolic clearance of sorafenib in HCC cells and enhanced the sensitivity of HCC cells to sorafenib (37). Knockdown of ABCG1 expression or inhibiting Wnt signaling has been reported to lead to reversal of saracatinib-induced oxaliplatin resistance in HCC. Blockade of IGF1-IGF1R signaling pathway is also closely associated with oxaliplatin resistance (38). In addition to the previously reported association of ferroptosis with sorafenib sensitivity, our experiments found that ferroptosis was also associated with oxaliplatin sensitivity. This not only expands our understanding of ferroptosis, but also expands our understanding of chemotherapeutic drug resistance, which is of great significance. In our current study, we provided a novel potential mechanisms underlying LINC01134 mediated enhancement of OXA resistance by focusing on possible molecular targets. In this work, we identified GPX4 as a critical target of LINC01134 in regulating oxaliplatin resistance through ferroptosis. Ferroptosis, a novel programmed cell death mode, is characterized *via* the accumulation of toxic lipid ROS in an iron-dependent manner (39). There are now already ferroptosis inducers (example: DHODH inhibitors) that are being developed as anticancer agents and are currently in clinical trials (40). And in many preclinical studies and clinical trials, inhibitors of ferroptosis (iron chelators DFP, N-acetylcysteine, etc.) have shown promising ability to alleviate parkinsonian symptoms (41). Recent studies have shown that the drug resistance of cancer cell can be addressed through ferroptosis (42). Glutathione Peroxidase 4 (GPX4) is the major enzyme preventing ferroptosis *via* converting lipid hydroperoxides to nontoxic alcohols (15). In addition, GPX4 can be used as a prognostic and typing marker for HCC (43). Therefore, inducing ferroptosis *via* inhibiting the GPX4 expression has become a treatment strategy to resolve chemoresistance problem of HCC (44). In our research, results showed that silenced LINC01134 initiate the ferroptotic cascade induced by RSL3 and enhanced the OXA sensitivity, which is consistent with the reports.

LncRNAs are involved in the development of a large number of cancers (45), by regulating numerous transcription processes (46). Studies have shown several lncRNAs have participated in ferroptosis *via* regulating GPX4 in various cells. Silenced lncRNA MEG8 can induce ferroptosis *via* significantly decreasing the expressions of SLC7A11 and GPX4 both in mRNA and protein level (47). Long non-coding RNA NEAT1 can regulate the sensitivity of ferroptosis depending on ACSL4 and GPX4 (48). Glutathione Peroxidase 4 (GPX4) is the major enzyme preventing ferroptosis *via* converting lipid hydroperoxides to nontoxic alcohols. In addition, GPX4 can be used as a prognostic and typing marker for HCC. Therefore, inducing ferroptosis *via* inhibiting the GPX4 expression has become a treatment strategy to resolve chemoresistance problem of HCC. Our previous study has shown that LINC01134/p62 axis plays an important role in regulating the OXA resistance of HCC (14), however, the effect of LINC01134 on OXA resistance cannot be completely explained by p62. Therefore, in our current study, we found LINC01134 is positively correlated with GPX4 and regulates OXA resistance in HCC through GPX4-mediated ferroptosis, the mechanism of which might compensate for the function of LINC01134 mediating OXA resistance. Additionally, we showed that LINC01134 enhanced OXA resistance dependent on another target, Nrf2. The role of Nrf2 in maintaining an appropriate redox homeostasis has been fully confirmed and it also has a pivotal role in mediating other crucial metabolic pathways including lipid metabolism, drug metabolism, apoptosis and so on (49). Research have shown that two ferroptosis-inducing agents, Erastin and RSL3, increased the ferroptosis level *via* restraining the cystine/glutamate transporter system xC-/xCT and GPX4 respectively, which are both downstream targets of transcription factor Nrf2. Studies have shown that knockout of GSTZ1 suppressed ferroptotic cell death *via* activating the Nrf2/GPX4 axis *in vivo* and *in vitro* (50). In addition, the GSK3β/Nrf2/GPX4 pathway has been reported to induce the OXA resistance of CRC by mediating KIF20A/NUAK1 activation (6). Our study revealed that LINC01134 knockdown in HepG2 and Huh-7 cells obviously increased sensitivity to OXA in two HCC cells and the sensitization was reversed by GPX4 re-expression. Since our study identified LINC01134/Nrf2/GPX4/axis can regulate OXA resistance, enhancing OXA sensitivity with GPX4 inhibitors would be a new and hopeful treatment strategy.

## Conclusions

In short, the results of this study revealed LINC01134, a novel negative regulator of ferroptosis, up-regulates GPX4 expression by enhancing the recruitment of the transcription factor Nrf2 to the GPX4 promoter, thereby increasing liver cancer resistance to OXA. Our research provides a reference for the targeted therapy of HCC.

## Materials and Methods

### Cell Culture, Plasmids, and Reagents

The human HCC cell line (HepG2) was gained from the American Type Culture Collection (Manassas). Huh-7 was a kind gift from Dr. Fan Feng at the General Hospital of the Chinese PLA. OXA sensitive and OXA resistant HepG2 cells were gained from the Department of Cellular Engineering Lab, Beijing Institute of Biotechnology. Cells were cultured in Dulbecco’s modified Eagle’s medium (Gibco) containing penicillin (100 U/ml), streptomycin (100 μg/ml) and 10% fetal bovine serum (Gibco) in a humidified atmosphere of 5% CO_2_ at 37°C. The eukaryotic expression vectors were constructed *via* inserting PCR-amplified fragments into pcDNA3.0 (Invitrogen). The luciferase reporter gene of GPX4 promoter was generated *via* inserting PCR-amplified promoter fragments from genomic DNA into the pGL4.0-Basic vector (Promega). Normal and mutated putative targets of Nrf2 on GPX4 were inserted into pGL4.0-Basic vector (Promega). SiRNAs of LINC01134 was synthesized by Biomed Company. The siRNAs target sequences for LINC01134 were listed in **Supplementary Table S1**. According to the manufacturer’s protocols, Lipofectamine RNAiMAX (ThermoFisher Scientific) and VigoFect (Vigorous Biotechnology) were used for transfections of siRNAs and plasmids. GPX4, Nrf2 and β-actin antibody were acquired from Proteintech. Erastin, RSL3 and AEM1 were acquired from Selleck. Sorafenib was acquired from MCE.

### Total RNA Extraction and Real-Time Quantitative PCR

According to the manufacturer’s instructions, total RNA was extracted from samples *via* TRizol reagent (Invitrogen). RNA was used to generate cDNA *via* Quantscript RT Kit (Tiangen). RT-PCR was conducted through the 20-μl reaction mixture which contains 10 μl 2 × TB Green (Takara), 9.0 μl diluted template, 0.5 μl sense primer and 0.5 μl antisense primer on CFX96 system (BioRad Laboratories Inc.). The relative expression of the target fold normalized to the corresponding control was calculated *via* the comparative cycle threshold values (2−ΔΔCt) method. The primers used in this experiment are shown in **Supplementary Table S2**.

### LncRNA *in Situ* Hybridization and Immunohistochemistry

58 human hepatocellular carcinoma cancer samples were obtained from the First Medical Center of Chinese PLA General Hospital. Fluorescent *In Situ* Hybridization (FISH) was conducted on paraffin tissue sections using human LINC01134 probes specific according to the manufacturer’s protocols (RiboBio). Immunohistochemistry (IHC) of formalin-fixed paraffin-embedded samples was conducted according to the manufacturer’s instructions (ZSGB-Bio). Briefly, tissue sections were deparaffinized, rehydrated and treated with 3% H2O2 for 15 minutes to restrain endogenous peroxidase activity. After recovery of heat-induced epitope in citrate buffer in microwave for 30 min, tissue samples were incubated overnight at 4°C with pre-diluted mouse anti-GPX4 (1:100) and rabbit anti-Nrf2 (1:100). After incubation with 100 μl biotin-labeled goat anti-mouse/rabbit IgG polymer, the signal was generated with 3, 3’-diaminobenzidine tetrachloride. Two pathologists blinded to patient profiles were asked to assess the expression independently. Receiver operating characteristic (ROC) curve analysis was utilized to evaluate the optimal cutoff value for IHC score. For correlation analysis, we defined score < 0.25, 0.25 ≤ score ≤ 0.75 and score > 0.75 as low, medium, and high LINC01134, GPX4 and Nrf2, respectively.

### Cell Viability and Colony Formation Assays

According to the manufacturer’s protocols, Cell Counting Kit-8 assay (Dojindo) was utilized to measure the cell viability. Cells were plated seeded in 96-well plates with the number of 3, 000 cells per well and then treated with oxaliplatin at concentrations of 0, 2, 4, 8, 16 μM. After 3 days, adding 10 μl of CCK-8 solution to the cultured cells in each well and incubate for 1 hour at 37°C. The OD values were then measured at 450 nm. Cells of colony formation assays were cultured in 35-mm plates with the number of 3, 000 cells per well and treated with OXA (8 μM) or not. The colonies were fixed with 4% paraformaldehyde for 30 minutes and stained with 1% crystal violet for 30 minutes. The visible colony count was counted by ImageJ software and the diameter of colonies more than 0.5 mm was counted.

### Assessment of Total ROS and Lipid ROS

Cells were seeded in 6-well plates with the number of 3 × 10^5^ cells per well. Next day, cells were treated with RSL3 for 8 h and incubated with 10 μM 2’, 7’-Dichlorofluorescin diacetate (total ROS) (Beyotime Institute of Biotechnology) or 10 μM C11-BODIPY581/591 (lipid ROS) (Invitrogen) for 30 min at 37 °C in the dark. Unincorporated dye was erased by PBS after 30 min incubation. The fluorescence intensity was analyzed through flow cytometry. And data analysis was conducted by FlowJo software or Graphpad Prism 8.0.

### Measurement of Glutathione (GSH) and Oxidized GSSG

According to the manufacturer’s instructions, the GSH and oxidized GSSG levels were tested through GSH and GSSG Assay kit (Beyotime Institute of Biotechnology). Glutathione (GSH) was measured by yeast-GSH reductase, 5, 5′-Dithio-bis (2-nitrobenzoic acid) and NADPH. The absorbance was recorded at 412 nm wavelength. The GSSG expression in the presence of 2-vinylpyridine was recorded through the same manner. Then the ratio of GSH: GSSG was calculated.

### Measurement of MDA

According to the manufacturer’s instructions, the Lipid Peroxidation MDA Assay Kit (Beyotime Institute of Biotechnology) was used to determine MDA levels. 150 μl of cell lysis buffer was added to cells and incubated for 10 min on ice. Supernatant from each tube of lysis buffer was collected *via* centrifugation at 12,000 g for 10 min under 4 °C.100 μl of the lysed sample was then mixed with 200 μl of malondialdehyde solution and incubated at 100 °C for 15 min avoiding from light. All mixtures were centrifugated at 1000 g for 10 min after cooling down to room temperature. 200 μl supernatant per tube was transferred into 96-well microplate to measure absorbance of the sample at 533 nm.

### Apoptosis Distribution Analysis

For apoptosis analysis, 1 × 10^6^ cells were seeded in 6 cm dishes. After the cells have adhered to the plate, they are transfected with siRNAs 48h before the addition of OXA (8 μM). Cells were collected for measurement after 3 days. According to the manufacturer’s protocols (Beyotime Institute of Biotechnology), the cells were labeled with Annexin V and propidium iodide. Samples were collected and tested by FACS calibur Flow Cytometer (Becton Dickinson).

### Luciferase Reporter Assay

To evaluate the effect of Nrf2 on the activity of GPX4 promoter, the GPX4 promoter reporter pGL4.0-GPX4 was co-transfected with β-galactosidase, empty vector, and Nrf2 overexpression vectors into cells. After 48 hours, the cells were collected. According to the manufacturer’s protocols (Promega), the activities of β-galactosidase and luciferase were analyzed. The activity of β-galactosidase served as an internal control of transfection efficiency.

### Chromatin Immunoprecipitation Analysis

Magna ChIP G Analysis Kit (Millipore) was used to perform ChIP assays according to the manufacturer’s instructions. Complexes were eluted from the primary immunoprecipitation by incubating with 10 mM DTT for 30 minutes at 37°C and diluted in ChIP buffer (2 mM EDTA, 1% Triton X-100, 150 mM NaCl, 20 mM Tris-HCl, pH 8.1). ChI DNA was analyzed *via* real-time PCR. The values were normalized to those of IgG or empty vector control.

### Statistical Analysis

All experiments were carried out in triplicate and repeated 3 times *in vitro*. The statistical significance of preclinical tests was evaluated *via* two-tailed Student’s t-test. All statistical tests were two-sided. SPSS 21.0 was used for statistical calculation. The difference was considered statistically significant (P < 0.05).

## Data Availability Statement

The original contributions presented in the study are included in the article/**Supplementary Material**. Further inquiries can be directed to the corresponding authors.

## Ethics Statement

The studies involving human participants were reviewed and approved by the Ethics Committee of Chinese PLA General Hospital. The patients/participants provided their written informed consent to participate in this study.

## Author Contributions

XK conceived the project and designed the experiments. AX, PZ, YH, and RL supervised the project. XK, YH, and SJ designed and performed the experiments. XK, YH, SJ, FH, HL, QZ, DL, and NC analyzed the data. XK, YH, and SJ wrote the manuscript. All authors contributed to the article and approved the submitted version.

## Funding

This work was supported by a grant from National Natural Science Foundation of China (No.81902440), Special program for cultivation of National Science Foundation for Excellent Young Scientists of PLA General Hospital.

## Conflict of Interest

The authors declare that the research was conducted in the absence of any commercial or financial relationships that could be construed as a potential conflict of interest.

## Publisher’s Note

All claims expressed in this article are solely those of the authors and do not necessarily represent those of their affiliated organizations, or those of the publisher, the editors and the reviewers. Any product that may be evaluated in this article, or claim that may be made by its manufacturer, is not guaranteed or endorsed by the publisher.

## Abbreviations

OS, disease free survival; HCC, hepatocellular carcinoma; lncRNA, long noncoding RNA; DFS, disease free survival; GPX4, glutathione peroxidase 4; OXA, oxaliplatin; PFS, progression free survival; ChIP, chromatin immunoprecipitation; FISH, fluorescence *in situ* hybridization; IHC, immunohistochemical staining; GSH, glutathione; GSSG, glutathione disulfide; Nrf2, nuclear factor E2-related factor 2; Scr, scrambled; RT-qPCR, reverse transcriptase-quantitative real time PCR; ROS, reactive oxygen species.
