# Supplementary material for: Silenced LINC01134 Enhances Oxaliplatin Sensitivity by Facilitating Ferroptosis Through GPX4 in Hepatocarcinoma
Source: Front Oncol. 2022 Jul 8;12:939605. doi: 10.3389/fonc.2022.939605 (PMC9304856; doi:10.3389/fonc.2022.939605)
Supplement: Supplementary file 3 [file DataSheet_3.pdf]

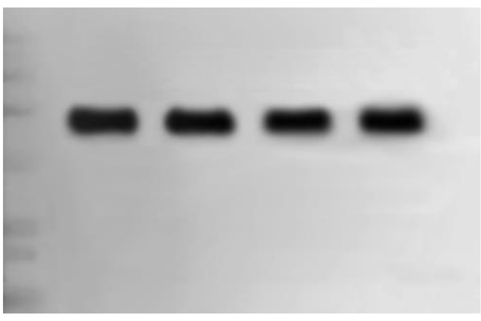
Figure 1F-β-actin

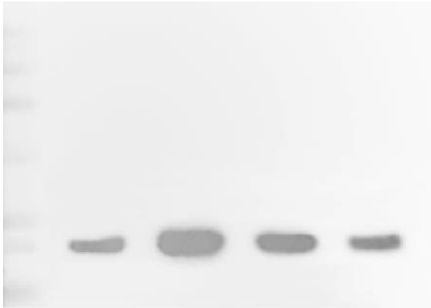
Figure 1F-GPX4

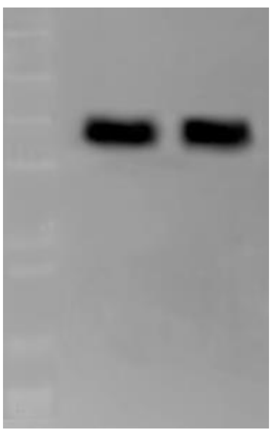
Figure 1G-HepG2-β-actin

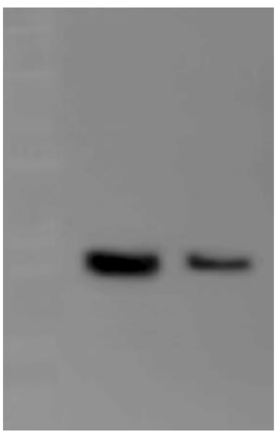
Figure 1G-HepG2-GPX4

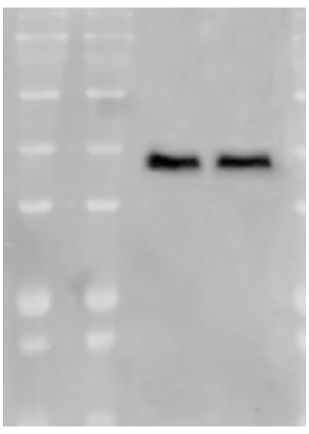
Figure 1G-Huh7-β-actin

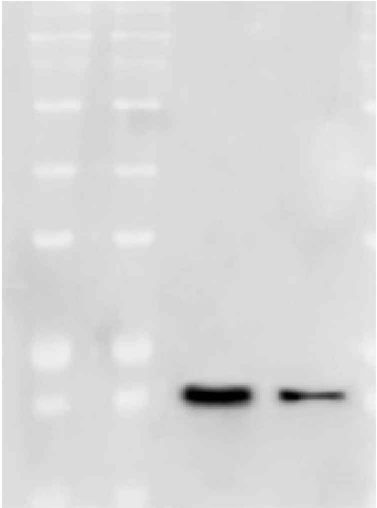
Figure 1G-Huh7-GPX4

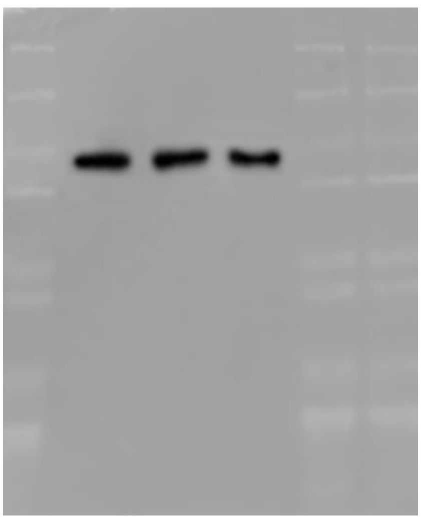
Figure 3A-β-actin

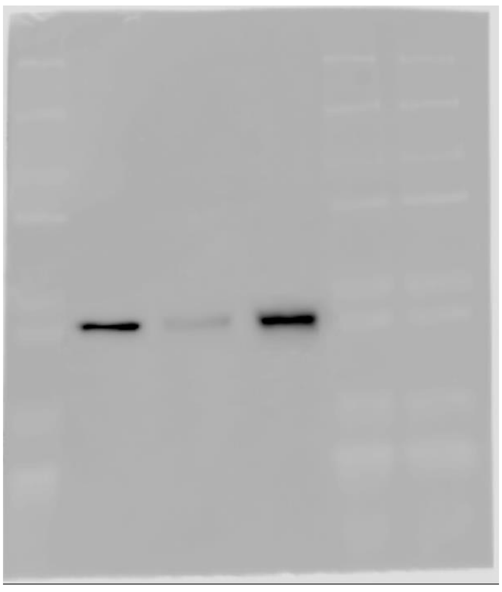
Figure 3A--GPX4

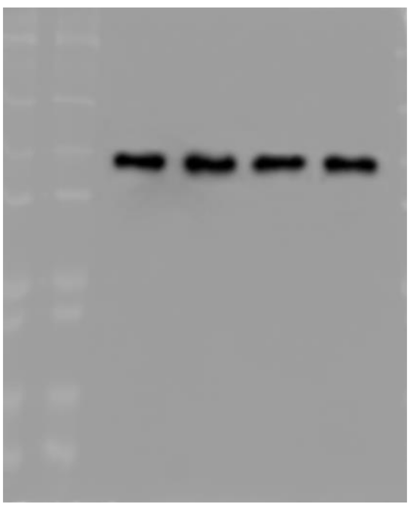

Figure 3C-β-actin

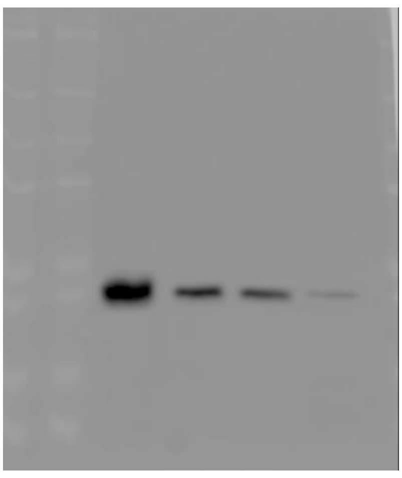

Figure 3C-GPX4

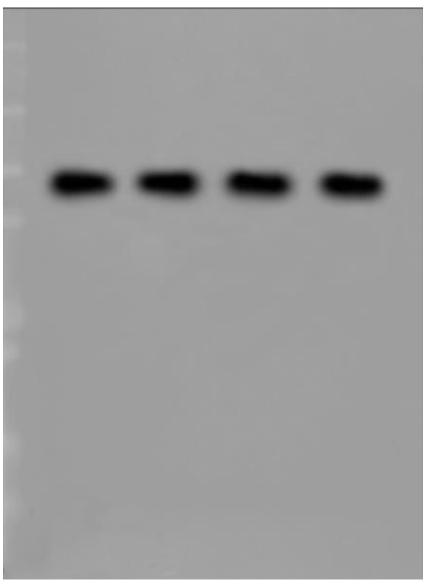

Figure 4E-β-actin

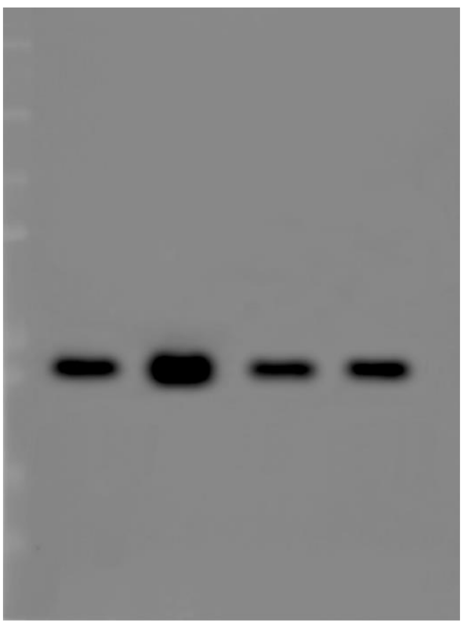

Figure 4E-GPX4

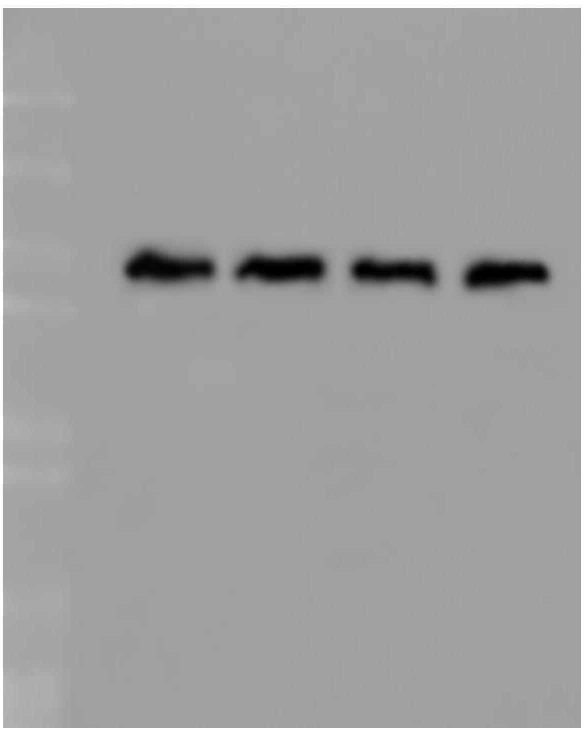

S-Figure 1I-β-actin

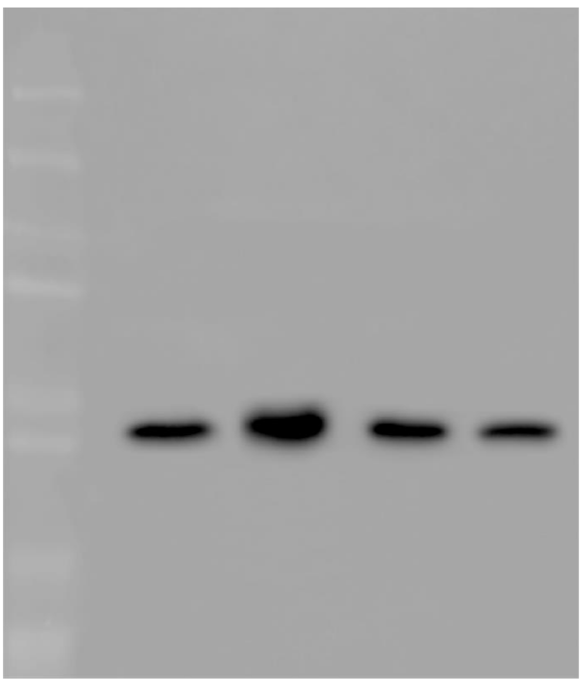

S-Figure 1I-GPX4

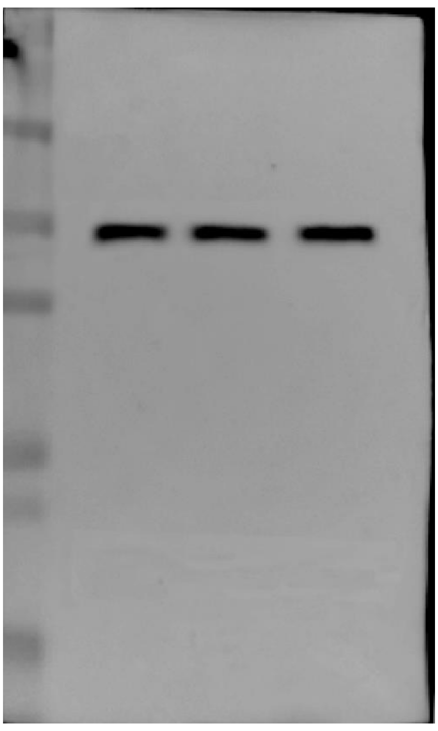

S-Figure 2A-β-actin

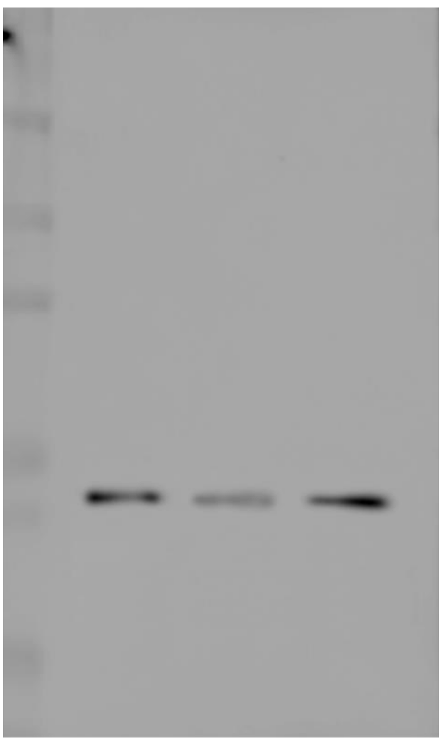

S-Figure 2A-GPX4

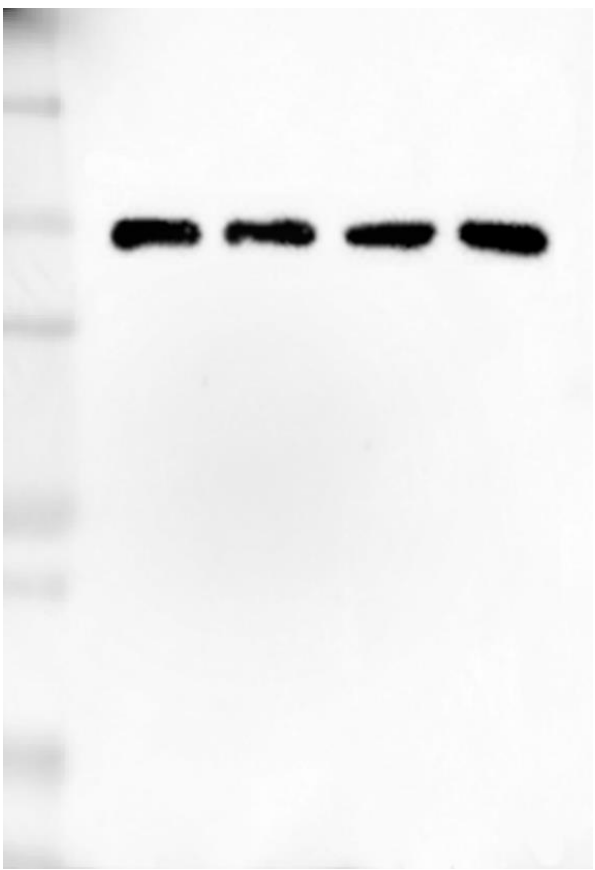

S-Figure 2C-β-actin

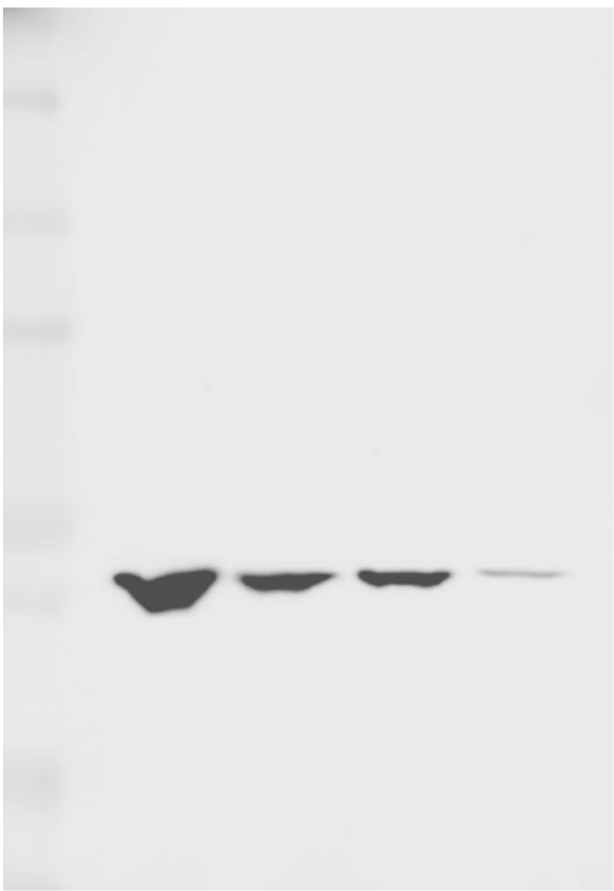

S-Figure 2C-GPX4

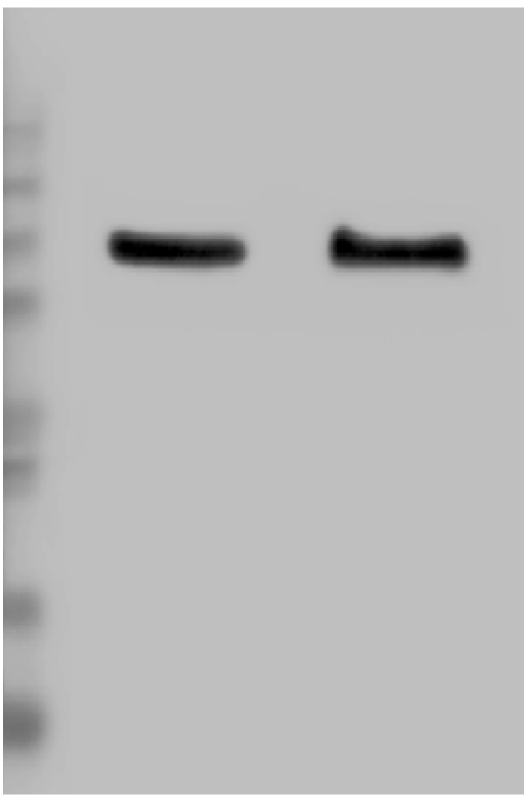
S-Figure 3B-HepG2-β-actin

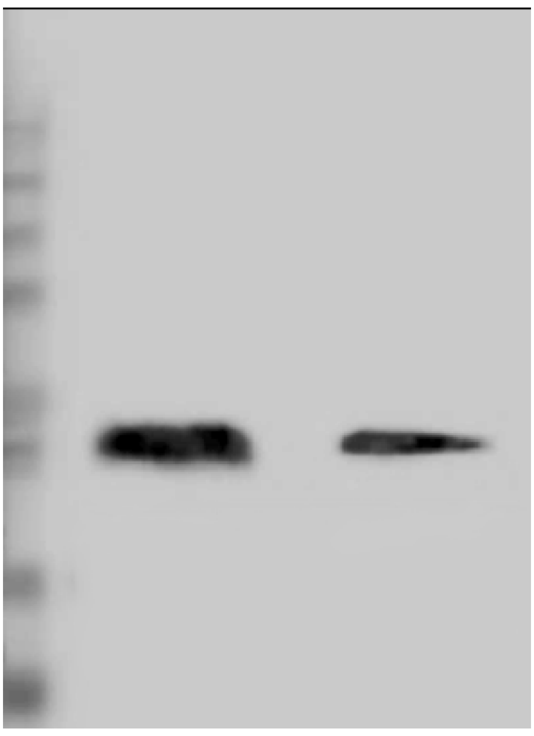
S-Figure 3B-HepG2-GPX4

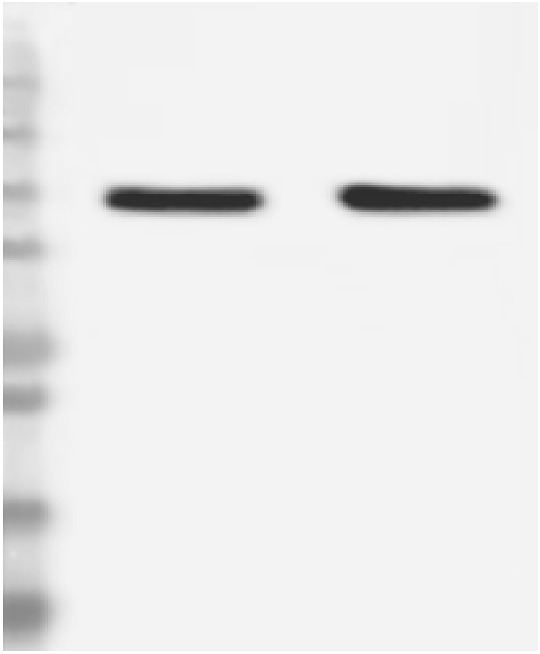
S-Figure 3B-Huh-7-β-actin

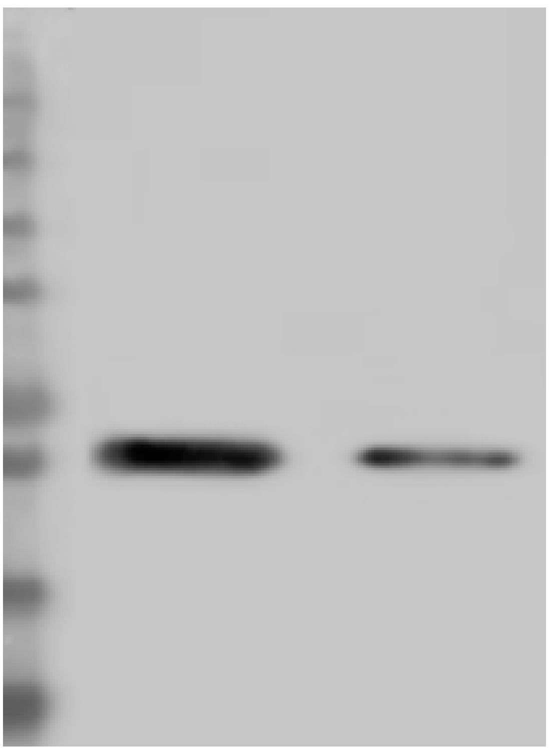
S-Figure 3B-Huh-7-GPX4
