# Supplementary material for: Silenced LINC01134 Enhances Oxaliplatin Sensitivity by Facilitating Ferroptosis Through GPX4 in Hepatocarcinoma
Source: Front Oncol. 2022 Jul 8;12:939605. doi: 10.3389/fonc.2022.939605 (PMC9304856; doi:10.3389/fonc.2022.939605)
Supplement: Supplementary file 4 [file DataSheet_4.pdf]

Figure S1

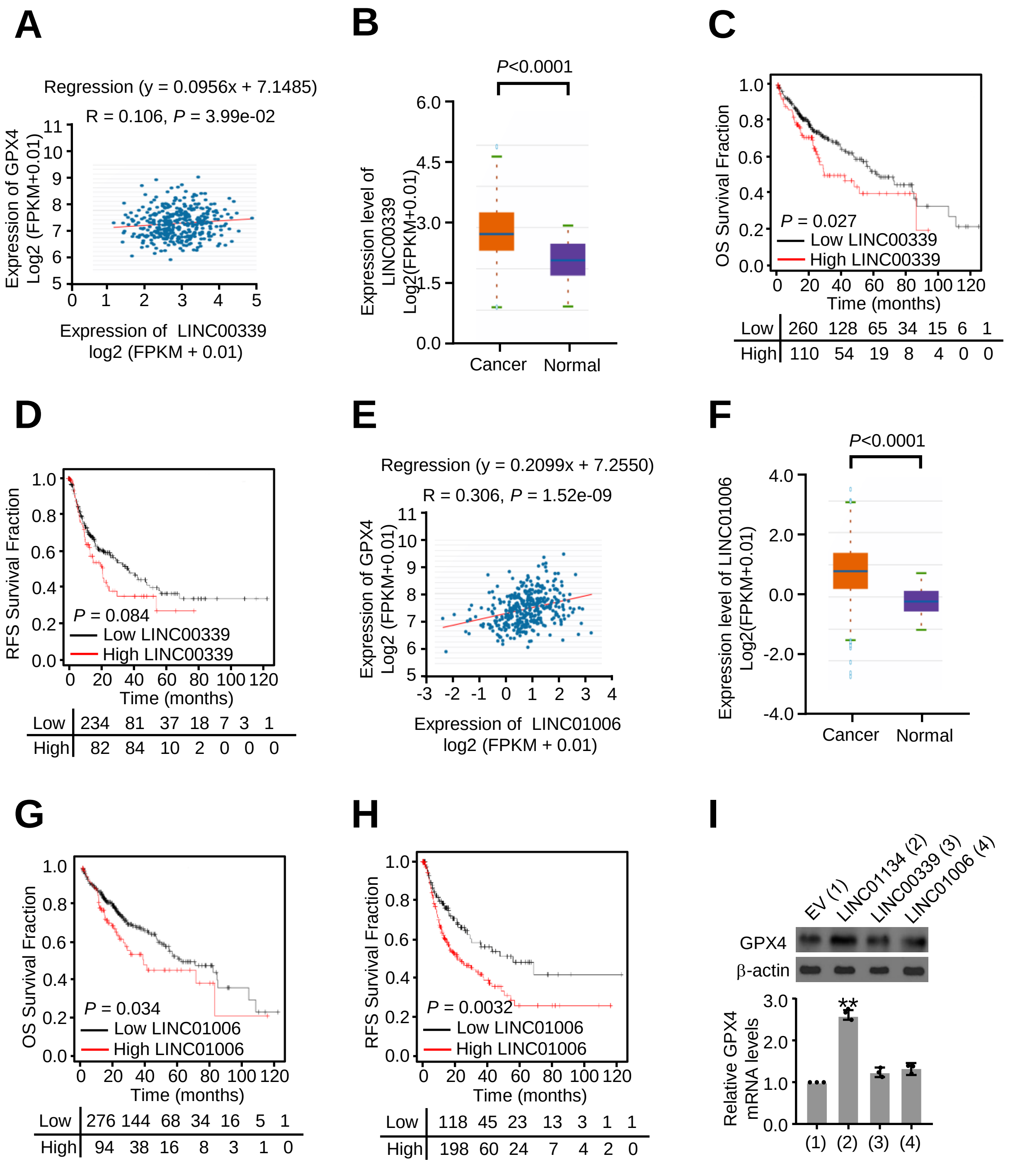

Supplementary Figure 1. Identification of LINC01134 as a lncRNA that is downregulated in HC and positively correlated with GPX4. (A) Pearson's correlation analysis of LINC00339 and GPX4 expression in liver cancer tissues (http://starbase.sysu.edu.cn/). (B) The LINC00339 expression levels between cancer tissues and normal tissues were compared by TNM plot (https://www.tnmplot.com/). (C and D) Kaplan-Meier analysis of the overall survival rate and recurrence-free survival (log-rank test, two sides) of HCC patients with low or high expression of LINC00339 (http://kmplot.com/analysis/). (E) Pearson's correlation analysis of LINC01006 and GPX4 expression in liver cancer tissues (http://starbase.sysu.edu.cn/). (F) The LINC01006 expression levels between cancer tissues and normal tissues were compared by TNM plot (https://www.tnmplot.com/). (G and H) Kaplan-Meier analysis of the overall survival rate and recurrence-free survival (log-rank test, two sides) of HCC patients with low or high expression of LINC01006 (http://kmplot.com/analysis/). (I) The protein and mRNA levels of GPX4 in Huh7 cells transfected with the empty vector, LINC01134, LINC00339 and LINC01006 expression vectors. (**P < 0.01).

Figure S2

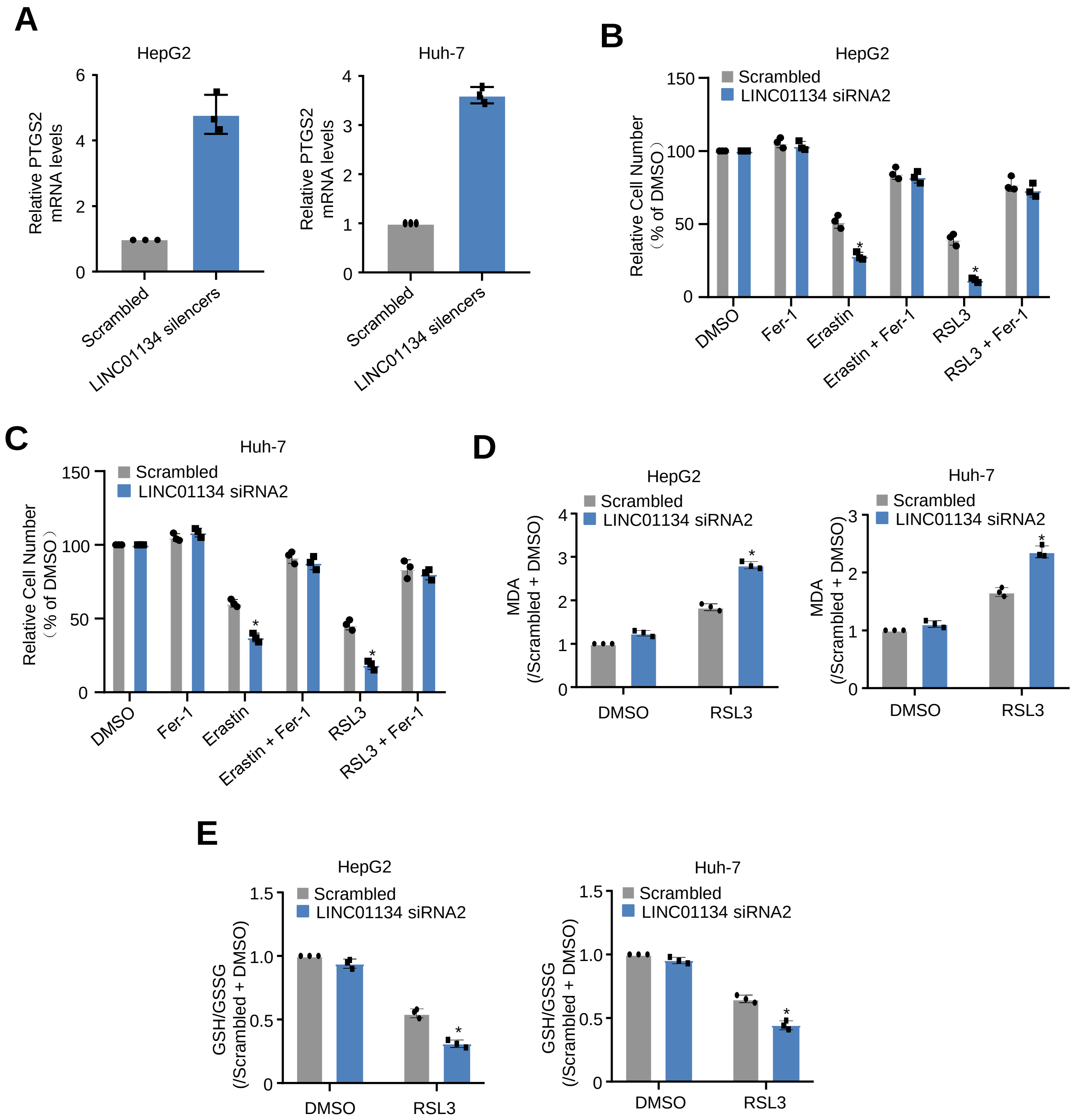

Supplementary Figure S2. Silenced LINC01134 enhances sensitivity of HCC cells to OXA and promotes RSL3-induced ferroptosise. (A). Huh-7 and HepG2 cells were transfected with scrambled or LINC01134 siRNA2. Relative PTGS2 mRNA levels were analyzed. (B-C) Huh-7 and HepG2 cells were transfected with scrambled or LINC01134 siRNA2 with or without treatment of RSL3 (2 μM), erastin (10 μM), and ferrostatin (2 μM). The histogram shows relative cell number measured by CCK-8 assays. (D-E) HepG2 and Huh-7 cells were transfected with scrambled or LINC01134 silencers with or without RSL3 (2 μM). Levels of Lipid Oxidation (MDA) (D) and GSH/GSSG (E) were analyzed. Data are shown as the mean ± SEM; n ≥ 3 independent experiments, two-tailed Student's t-test: *P < 0.05.

Figure S3

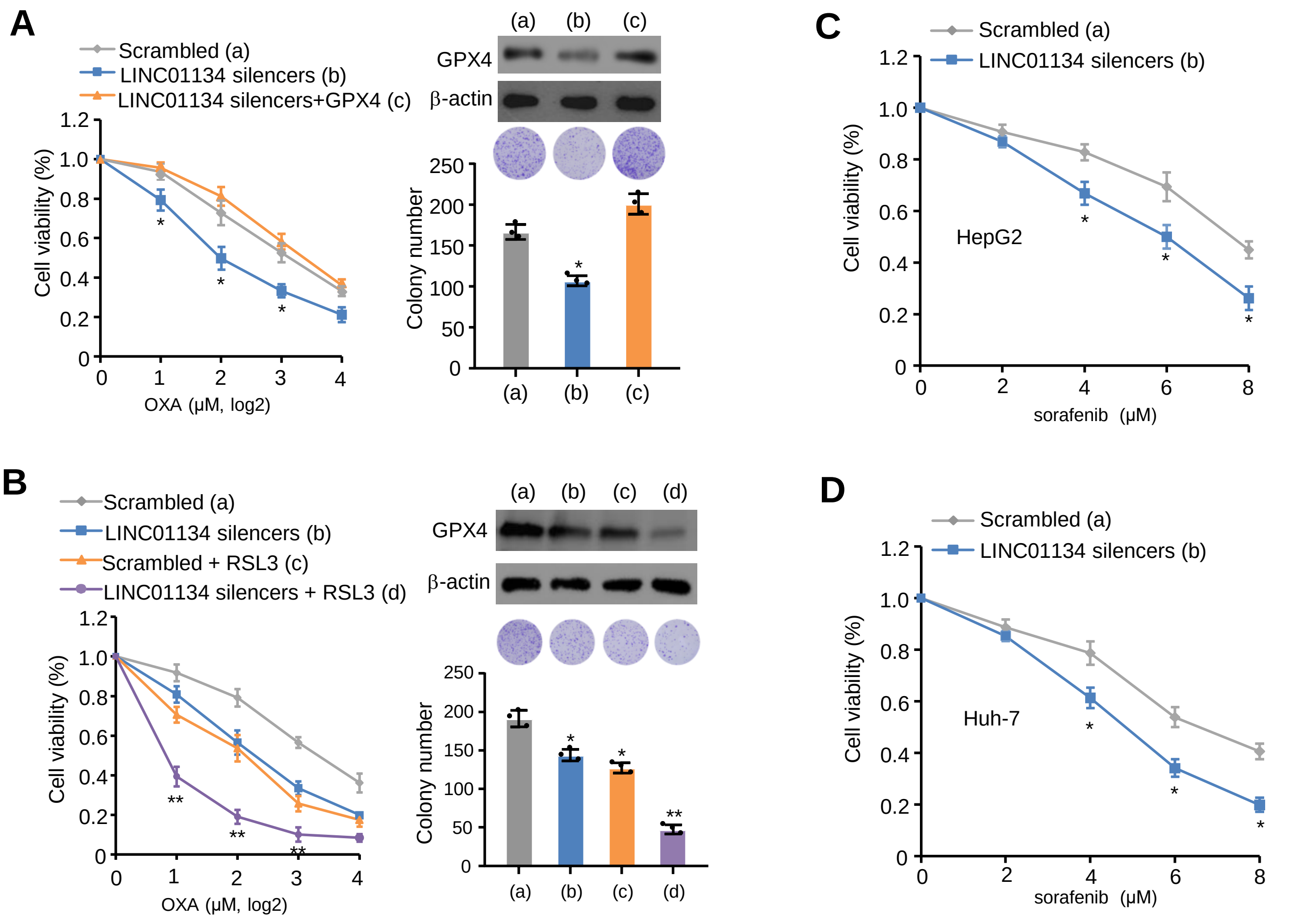

Supplementary Figure S3. Silenced LINC01134 downregulates OXA resistance through GPX4 pathway in Huh-7 cell line. (A) Huh-7 cells were transfected with scrambled, LINC01134 silencers, or LINC01134 silencers plus pcDNA3.0-GPX4. Relative cell number was determined by CCK-8 assays. Representative immunoblot shows the GPX4 expression. Representative image of colony formation assay shows the colonies in plates. The histogram shows colony number. (B) Huh-7 cells were transfected with scrambled, LINC01134 silencers, with or without RSL3 treatment. Relative cell number was determined by CCK-8 assays. Representative immunoblot shows the GPX4 expression. Representative image of colony formation assay shows the colonies in plates. The histogram shows colony number. (C) HepG2 cells were transfected with scrambled, LINC01134 silencers. Relative cell numbers were determined at different concentrations of sorafenib by CCK-8 assays. (D) Huh-7 cells were transfected with scrambled, LINC01134 silencers. Relative cell numbers were determined at different concentrations of sorafenib by CCK-8 assays

Figure S4

A

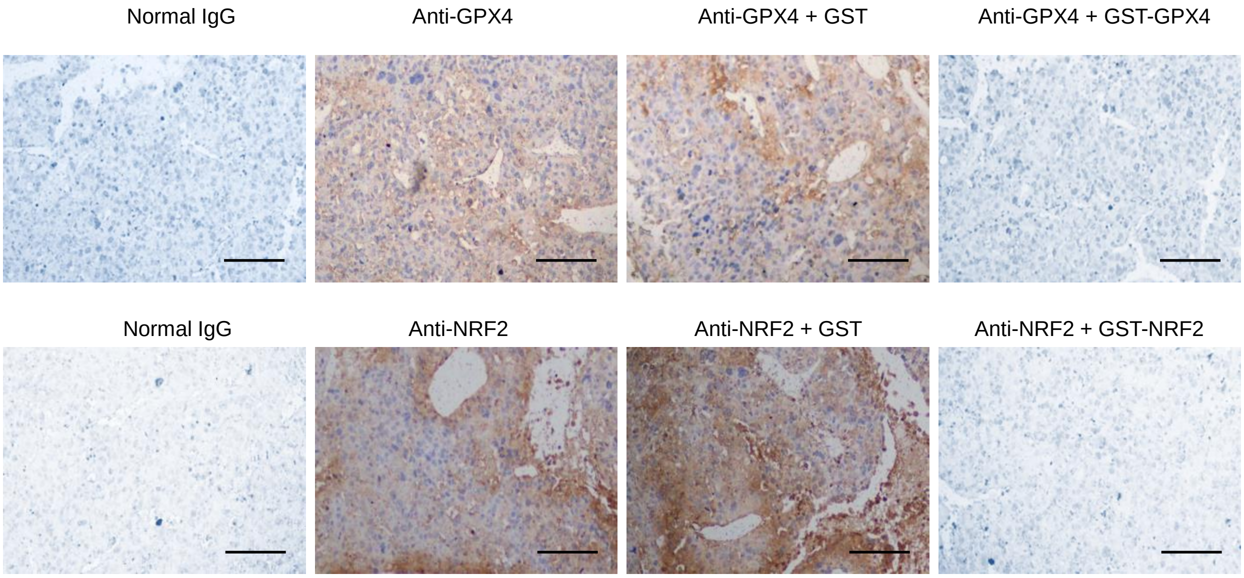

B

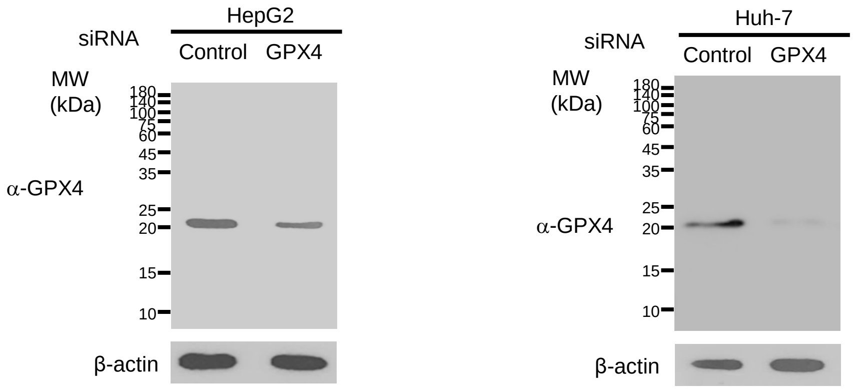

Supplementary Figure S4. Identification of the specificity of the antibody. (A) IHC staining of thyroid cancer specimens incubated with normal IgG and anti-GPX4 (or anti-Nrf2). To confirm the antibody specificity, anti-GPX4 (or anti-Nrf2) was pre-incubated with the indicated recombinant GST or GST-GPX4 (or GST-Nrf2) applied to the tissue for 1 hour. Scale bar, 50 μm. (C) Western blot analysis of lysates from HepG2 or Huh-7 cells infected with control siRNA or GPX4. The data shown is the average ± SD of three measurements, which has been repeated 3 times and the results are similar. **P < 0.01 versus case (1).

Figure S5

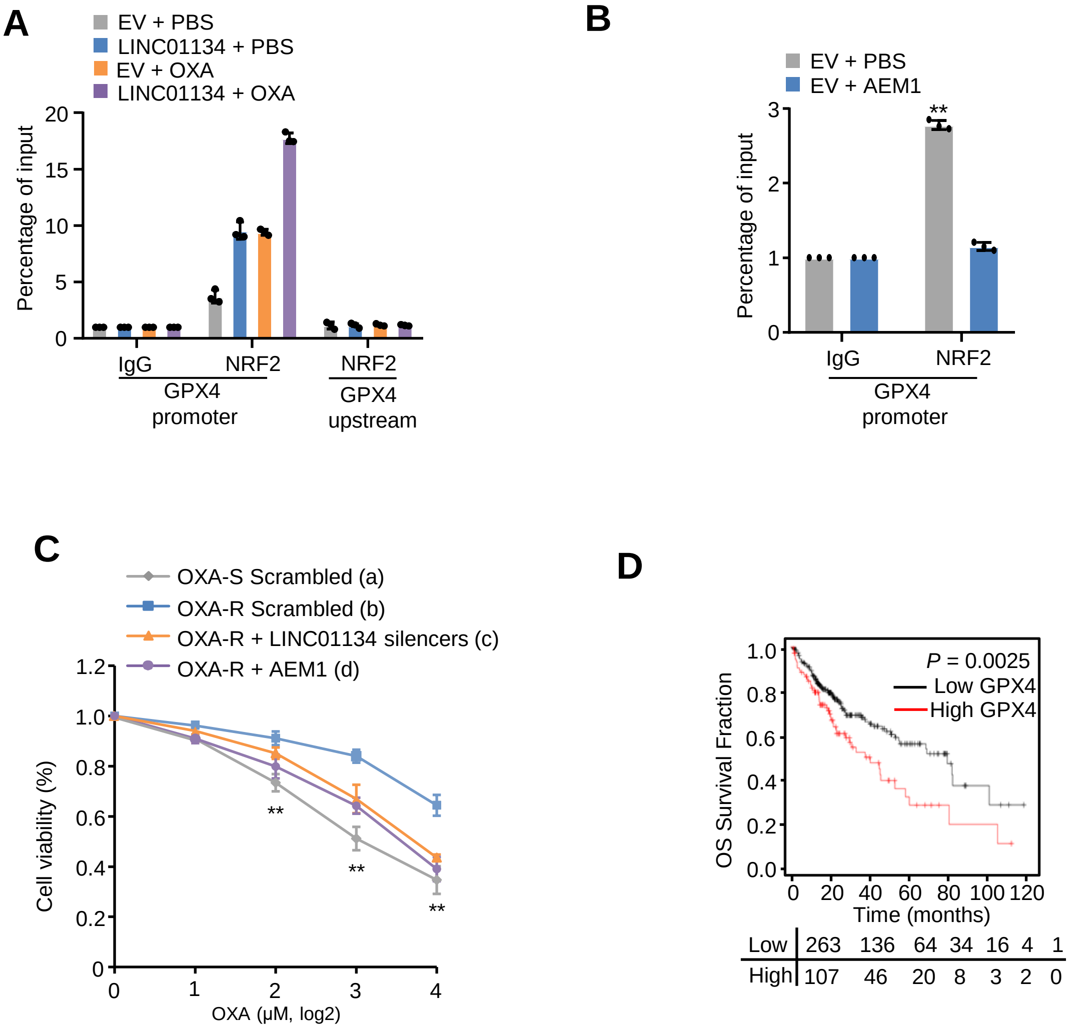

Supplementary Figure S5. (A) ChIP analysis for Nrf2 occupancy on the GPX4 the promoter upstream or promoter in Huh-7 cells transfected with LINC01134 and treated or not with OXA. (B) ChIP analysis for Nrf2 occupancy on the GPX4 the promoter promoter in HepG2 cells treated or not with AEM1. (C) OXA sensitive and OXA resistant cells were transfected with scrambled, LINC01134 silencers,and treated with AEM1. Relative cell number was determined by CCK-8 assays. (D) Kaplan-Meier analysis of the overall survival rate (log-rank test, two sides) of HCC patients with low or high expression of GPX4 (http://kmplot.com/analysis/).
