# Supplementary material for: Silenced LINC01134 Enhances Oxaliplatin Sensitivity by Facilitating Ferroptosis Through GPX4 in Hepatocarcinoma
Source: Front Oncol. 2022 Jul 8;12:939605. doi: 10.3389/fonc.2022.939605 (PMC9304856; doi:10.3389/fonc.2022.939605)
Supplement: Supplementary file 5 [file DataSheet_5.doc]

**Table S1. The cDNA target sequences of siRNAs**

| **Gene** | **Target sequence (5’→3’)** |
| --- | --- |
| LINC01134 (siRNA1)  LINC01134 (siRNA2)  LINC01134 (siRNA3)  LINC01134 (siRNA4)  LINC01134 (siRNA5)  LINC01134 (siRNA6) | GACACGAGAACCAGCCAAGA  TTGTAGCTTCTGTGTTCGCC  GGGAAAGGCCTGAAAGTAAA  TCATCCATCTGATCATCAA  GCATCCACTCATTCACTCA  GTGTTCGCCATATTTGAAA |

Table S2. Primers used for real-time PCR

| **Gene** | **Species** | **Forward (5’→3’)** | **Reverse (5’→3’)** |
| --- | --- | --- | --- |
| LINC01134  GPX4  β-actin | Human  Human  Human | GGCTTACCGTTTCCCCAGGG  GAGGCAAGACCGAAGTAAACTAC  ATCACCATTGGCAATGAGCG | CAGAATGGCAGGAATCAAGG  CCGAACTGGTTACACGGGAA  TTGAAGGTAGTTTCGTGGAT |

Table S3. Primers used for ChIP

| **Gene** | **Forward (5’→3’)** | **Reverse (5’→3’)** |
| --- | --- | --- |
| GPX4-promoter | GTCCCAGCTACTCGGGAAG | GCAGAAAAGTGTCCCCAAC |
| GPX4-upstream | TCAACCCGGCCCTGAGTC | ATCCGCCTGCCTCGGCC |

**Table S4. Information of the chemotherapy regimens of the patients**

| **OXA** | **Treatment** | **Number of patients** |
| --- | --- | --- |
| **+** | OXA + 5-FU + Calcium folinate | 29 |
|  | 5-FU + Cisplatin | 11 |
| - | 5-FU + Epirubicin | 3 |
|  | 5-FU + Mitomycin | 1 |
|  | 5-FU + Doxorubicin | 14 |
